# Olfactometric and Chemical Characterisation of Gaseous Emission from Crude Oils

**DOI:** 10.3390/molecules30051136

**Published:** 2025-03-01

**Authors:** Elisa Polvara, Vittoria Legnani, Marzio Invernizzi, Selena Sironi

**Affiliations:** Department of Chemistry, Materials and Chemical Engineering “Giulio Natta”, Politecnico di Milano, Piazza Leonardo da Vinci 32, 20133 Milano, Italy; elisa.polvara@polimi.it (E.P.); selena.sironi@polimi.it (S.S.)

**Keywords:** odour emissions, volatile organic sulphur compounds (VOSCs), dynamic olfactometry, TVOC, odorous potential

## Abstract

This study focuses on the olfactometric and chemical characterisation of gaseous and vapour emissions from different crude oils. To investigate this topic, laboratory experiments were set up to obtain comparable gaseous samples: they were estimated in terms of odour concentration (C_od_), via dynamic olfactometry, and chemical-specific characterisation. It was found that, even if considered similar in regard to physical properties and chemical composition, the gaseous emissions of different crude oils are significantly different in terms of odorous potential. The observed discrepancy appears to be associated with the presence of volatile organic sulphur compounds (VOSCs), and the highest values of C_od_ were found in samples containing mercaptans and sulphides. In addition, from the conducted comparison, it appeared that crude odorous potential, in terms of C_od_, is not strictly linked to the quantity of the volatile organic compounds (VOCs), H_2_S concentration, or a priori knowledge of the percentage of elemental sulphur in the crude; on the contrary, the presence of volatile organic sulphur compounds in the gaseous emissions is the most influential parameter for the odour potential of this matrix.

## 1. Introduction

In the oil extraction and refinement field, the handling of crude oil is one of the main sources of volatile organic compounds (VOCs). The loading and unloading operations of the oil tankers and their storage the tanks in coastal depositories or refineries represent a major contribution to global VOC emission inventory [1,2]. Indeed, the entire process (from extraction to final use) is connected with the emission of VOCs, particularly BTEX and light aliphatic hydrocarbons, into the atmosphere [3,4]. 

The odour harassment connected with the entire crude oil refinery process is a well-known problem, and different scientific papers and regulatory agencies have highlighted the topic and delved into the mitigation and the control of odour emissions from this process [3,5,6,7,8]. However, although this theme has been known for some time and the characterisation of the crude oil liquid matrix has been investigated [9,10,11,12], the odorous potential of crude oil gaseous emissions remains very poorly investigated. Indeed, crude oils comprise a complex and variable matrix, whose chemical composition and correlated gaseous emissions are not usually known a priori [13,14]. Indeed, in the oil-refining field, this matrix is characterised from a physical point of view (e.g., density, viscosity, content of elemental sulphur and nitrogen), and crude oils are generally considered as a unique category in terms of diffuse emissions, with similar VOC emission properties [11]. 

Crude oils are a mixture of hydrocarbon compounds, such as paraffins (~30% m/m), naphthenes (~40% m/m), and aromatics (~25% m/m), plus small amounts of organic compounds of sulphur, oxygen, and nitrogen (≤ 5% m/m) [15]. Moreover, traces of metallic compounds such as vanadium, nickel, and sodium may be present. The proportions of these elements are generally 84.5% carbon, 13% hydrogen, 1–3% sulphur, and less than 1% each of nitrogen, oxygen, metals, and salts [15,16,17,18]. Therefore, a specific crude oil may contain a large number of compounds and isomers that are not easily identifiable or quantifiable individually. In addition, the elemental composition of crude oils depends on the type and origin of the crude, i.e., the geological origin and processes during formation and migration can influence the final composition of crude oils [19,20]. 

Despite this complexity, the scientific and technical literatures describe limited knowledge about crude oils. 

As previously noted, due to the great emission potential of crude-handling processes, in recent years, the attention toward VOC releases from these activities has been increasing due to the increasing interest in their environmental and health impacts on workers and the resident population [12,21,22,23,24,25,26,27]. Another important issue deriving from the emissions of VOCs is the odour nuisance, which often raises concern amongst the population because of its association with potential health effects and to environmental stress correlated with odorous exposure [28,29,30,31,32]. Even if odour nuisance does not represent a direct problem for human health, prolonged exposure to odours may cause reversible negative effects that may adversely affect the quality of life, both in terms of physiological and psychological outcomes, such as headaches, eye/throat irritation, or stress and emotional distress [31,33].

Despite the growing interest in VOCs emissions, few works have been published in the scientific literature regarding the chemical characterisation of gaseous emissions from crude oil storage and handling. In addition, the available studies regarding the chemical characterisation of crude oils, to the best of our knowledge, did not investigate their odorous potential.

Concerning crude oil tanker emissions, a recent study [13] exploited simulation software to model and identify significant VOCs in hydrocarbon vapour emissions during crude oil loading operations. The study, based on assumptions regarding the compositions of the three different crude oils, was more focused on the influence of operating conditions (i.e., temperature, pressure, and tank level) on vapour emissions rather than on their chemical compositions. Actually, the simulation provided only a rough qualitative composition of the vapours, i.e., the lower boiling point VOCs are estimated as the most present, benzene is the only identified aromatic compound, cyclopentane is the most significant naphthenic component, and pentane is the main paraffinic component. Moreover, the input data regarding compositions are just hypotheses, limiting the robustness of the method and the reliability of the conclusions. 

Crude oils gaseous emission was also investigated in a second study [34], in which a gas chromatograph coupled with a flame ionization detector (FID) was used, analysing hydrocarbons emitted from crude oil tankers. It was concluded that, on average, the vapours released by crude handling are composed of 13% hydrocarbons, i.e., methane, ethane, propane, butane, pentane, hexane, and a smaller number of heavier components: amongst all of these, propane and butane constitute the major components. The other components include 70% nitrogen, 10% carbon dioxide, 5% oxygen, and 2% other compounds, such as hydrogen sulphide. 

In a third study [35], an experimental investigation of crude oil storage tank emissions was conducted, characterizing the VOCs in the headspace of a typical liquid oil storage tank battery. Through gas chromatography–mass spectrometry (GC–MS) analysis, the main constituents identified were alkanes (from propane to nonane, with numerous isomers), only a few aromatics (benzene, toluene, and p-xylene), and CO_2_. However, despite the importance of the cited studies, the correlation between the chemical composition of crude oil emissions and their odorous potential was never investigated. In addition, no studies were conducted to assess the differences in emissivity in terms of chemical composition and odour among different types of crude oil. Therefore, due to the lack of information available in this field, this paper is a first attempt to deepen the knowledge of the atmospheric emissions, both in terms chemicals and odour, of different types of crude oils to obtain a detailed composition of the possible emissions derived from oil handling. In addition to this aim, an additional, and more daring, objective of this study is to correlate the obtained odour potential with the resulting chemical composition to find the parameters mainly responsible for odour harassment and to provide useful information for their abatement/containment.

## 2. Results and Discussion

### 2.1. C_od_ and TVOC Measurement

**Figure 1:**
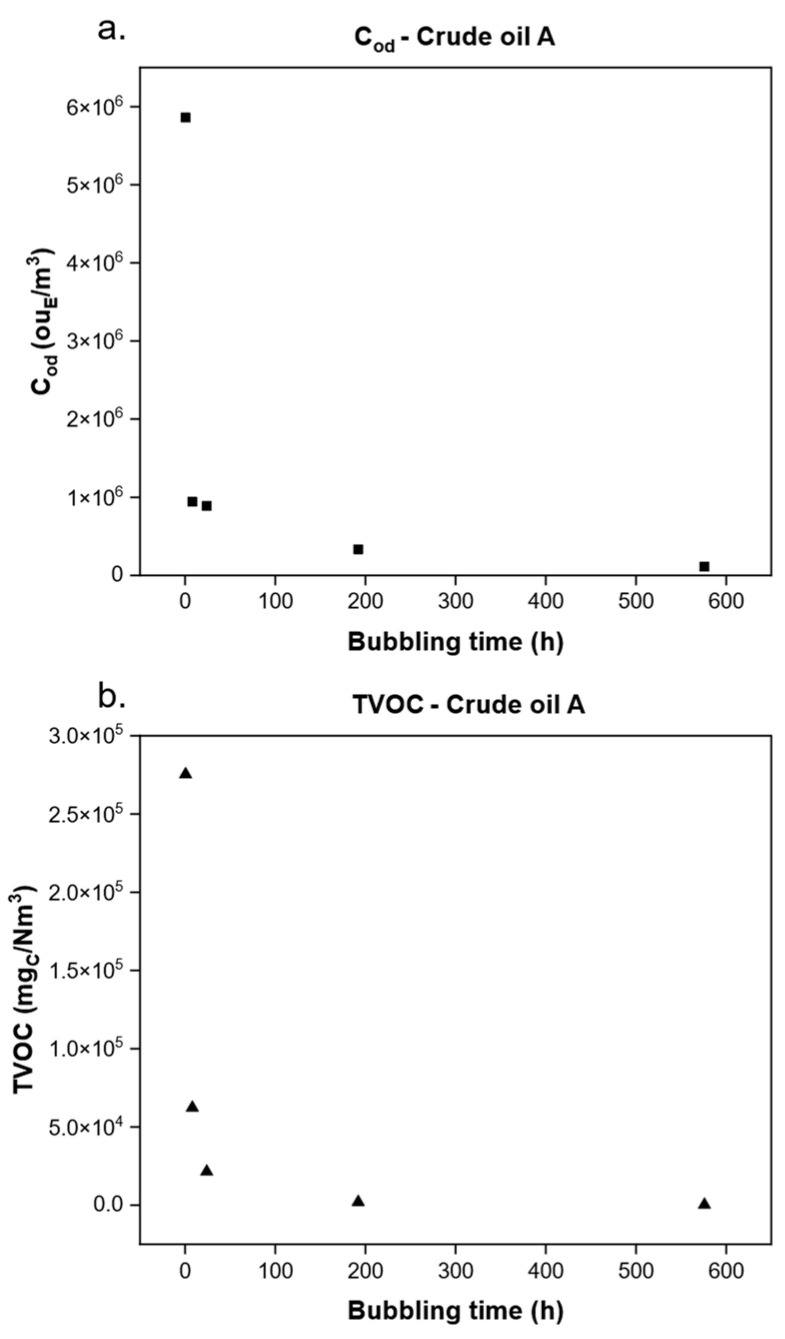
(**a**) Trend along time of C_od_; (**b**) TVOC of crude oil A.

First, a preliminary evaluation of the emissivity along the overall bubbling time was conducted to investigate the concentrations’ trend during the aeration, and consequent evaporation, of the liquid. Therefore, the evolution of C_od_ and TVOC over time (i.e., at five different sampling times: 30 min; 8, 24, 192, and 576 h) was examined for all the crudes. Due to the similarity in the obtained trends between the tested crude oils, only the outcome for crude oil A is reported in Figure 1.

**Figure 2:**
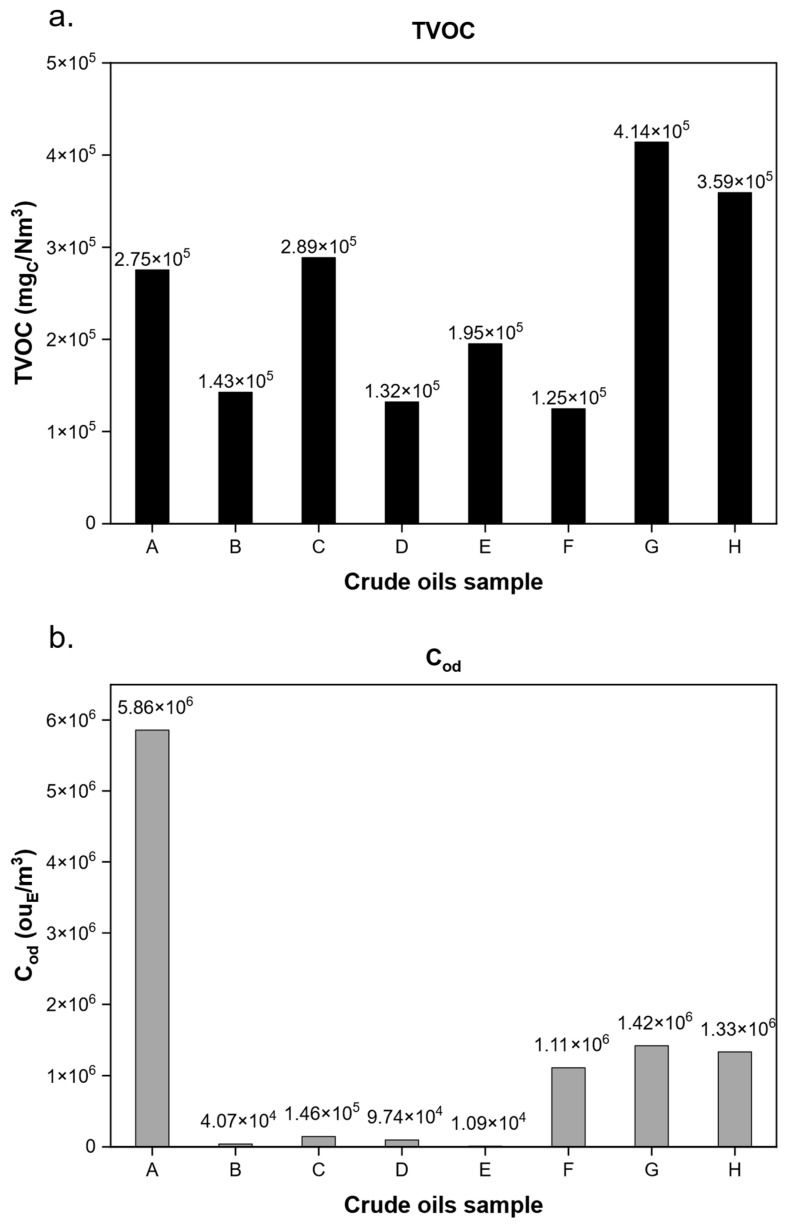
(**a**) TVOC and (**b**) C_od_ of first samples (30 min) obtained by different crude oils.

As expected, as the aeration time proceeds, both the C_od_ and TVOC of the gaseous samples decrease and, especially in the first portion of bubbling, the majority of volatile compounds is lost from the liquid, as can be observed by the decreasing in TVOC from 2.8 × 10^5^ to 6.2 × 10^4^ mg_C_/Nm^3^ within the first 30 min. Note that this trend is common to all the crude oils tested in the study: during the bubbling of crude oil, a steep decrease, both in terms of odour (C_od_) and VOCs (TVOC), was observed between the first two samples. After this preliminary observation, a direct comparison of each crude oil was conducted. Figure 2 depicts a comparison of TVOC and C_od_ detected in each first sample obtained from each crude oil. The comparison was conducted on these samples, collected at 30 min into each bubbling trial because, as previously mentioned **(**Figure 1), these samples were the richest in terms of both TVOC and C_od_.

As can be noted from Figure 2a, the TVOC values of the different crude oils were rather similar among each other, showing the same order of magnitude and ranging, at most, by a factor of three between the minimum and maximum. On the contrary, by observing Figure 2b, a much greater variability in terms of C_od_ was obtained: the C_od_ values span over two orders of magnitude among the analysed crude oil samples. Thus, despite these two parameters showing a similar trend concerning the flushing time (Figure 1), the C_od_ obtained from different crude oils varied drastically. Therefore, in order to ascertain the reason for this discrepancy, a comprehensive chemical characterisation was conducted for the purpose of identifying volatile molecules present in the crude oil vapours.

### 2.2. Chemical Analyses: Identification and Quantification of VOCs

#### 2.2.1. Speciation and Quantification of VOCs: GC–MS/FID/PFPD

**Figure 3:**
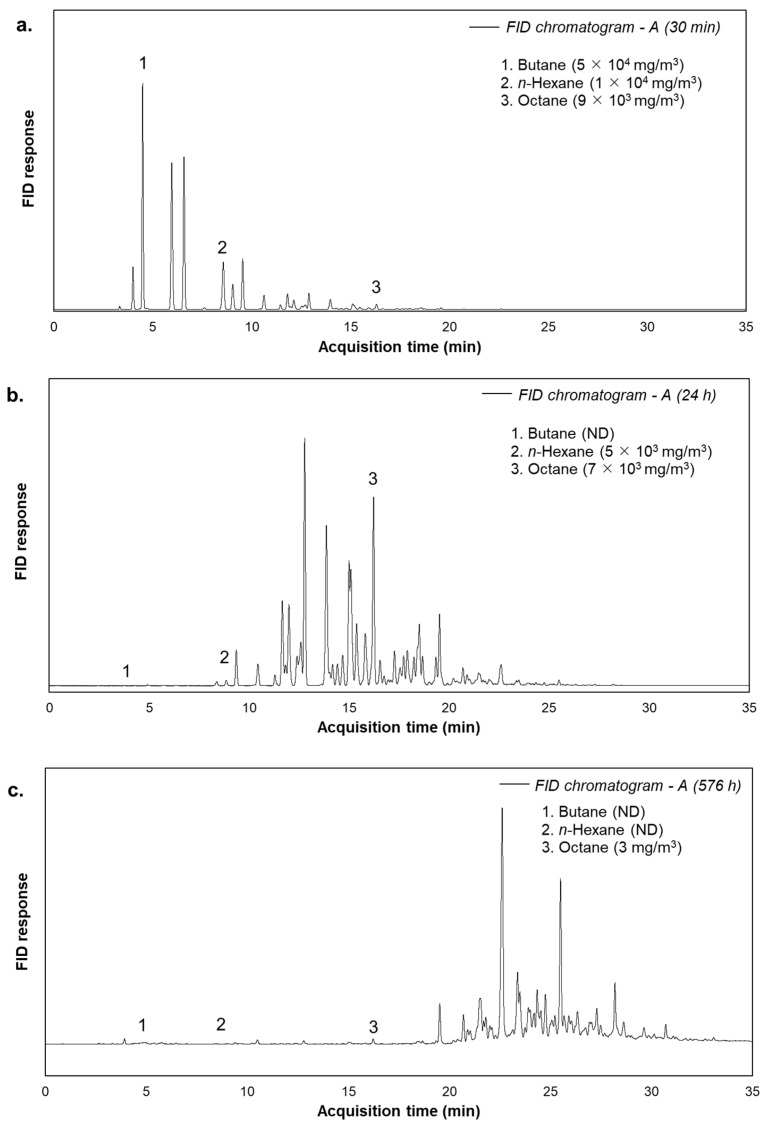
GC-FID chromatograms for crude oil A, sampled at 30 min (**a**), 24 h (**b**) and 576 h (**c**). ND stands for Not Detected. Detailed quantifications are reported in Supplementary Materials (Tables S1–S8).

A preliminary GC investigation was conducted to deepen the TVOC downward trend, obtained by FID analysis, along the bubbling time (Figure 1). Figure 3 reports the GC-FID chromatograms of crude oil A, corresponding to the first sampling time (30 min), an intermediate time (24 h), and the final time (576 h). In addition, in the reported chromatograms, the concentrations of three single paraffins (butane, hexane, and octane), significant constituents of the emission, are singularly reported.

For all the crude oils investigated, GC-FID chromatograms showed that, at the beginning of the bubbling, the predominant compounds emitted are the most volatile, with a low retention time (e.g., isobutane, butane, pentane). Proceeding with the bubbling time, the predominant species shift to less volatile types (e.g., decane-4-methyl, undecane, dodecane), eluted at a higher retention time. In addition, even individual VOC concentrations, as reported in Figure 3, present a general decrease, consistent with expectations and in line with the TVOC trend (Figure 2). All the observations could be justified by the differing volatility of the observed species, i.e, along with the bubbling, very volatile compounds tend to evaporate easily due to their low molecular mass and high vapour pressure. As a result, the most volatile compounds are released abruptly during the first bubbling period, with very high concentrations. Conversely, the less volatile compounds are emitted more slowly and in lower concentrations than the results for the more volatile types. After only one day of bubbling, some of the most volatile molecules (e.g., butane) are used up, while heavier types are still present in the stripped gas. At the end of the bubbling trial, even intermediate chain hydrocarbons (e.g., hexane) are absent in the gas phase, and the heaviest molecules (e.g., octane) are detected at very low concentrations.

In addition, to understand the variability of observed C_od_ amongst different crude oils (Figure 1), an extensive investigation of the composition of gas samples, in terms of the qualification and quantification of compounds, was conducted using GC–MS/FID/PFPD instrumentation. The identification and quantification obtained for the first sample (30 min, the most concentrated sample in terms of TVOC and C_od_) of all the eight investigated crude oils are reported in the Supplementary Materials, Tables S1–S8. To evaluate the differences in odour potential between the crude oils, the study concentrated on examining the gaseous sample obtained at 30 min, as the decrease in odour is evident throughout the bubbling.

In terms of single VOCs, detected via GC-FID, and TVOC, it is not possible to observe, from the experimental data, a clear explanation for the difference in C_od_ observed among the crude oils. 

**Figure 4:**
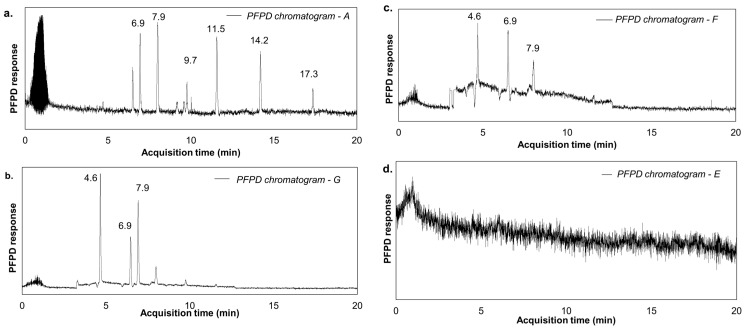
GC-PFPD chromatograms of crude oil A (**a**), crude oil F (**b**), crude oil G (**c**), and crude oil E (**d**). Detailed quantifications are reported in the Supplementary Materials (Tables S1–S8).

On the contrary, due to the combination of an additional detector (PFPD, specific for sulphur compounds), it was possible to notice a significant variation amongst the analysed crude oil VOSCs, in terms of organic sulphur compounds. In Figure 4, four different GC-PFPD chromatograms, detected for the first samples (30 min, the most concentrated sample in terms of TVOC and C_od_), obtained from four different crude oils, are presented.

The chromatograms show a great variability in the content of VOSCs released in the gas phase. Indeed, crude oil A was mainly the richest in VOSCs. The identified molecules included ethanethiol, dimethyl sulphide, 1-propanethiol, 2-propanethiol, thiophene, dimethyl disulphide, and methyl ethyl disulphide. An intermediate outcome was obtained for crude oils F and G, in which only methanethiol, dimethyl sulphide, and 2-propanethiol could be identified. On the contrary, for crude oil E, no peaks were identified in correlation with the presence of sulphur compounds (Figure 4d), meaning that sulphur compounds were not detected (<LoQ = 0.03 mg_S_/m^3^).

#### 2.2.2. Quantification of Hydrogen Sulphide

In parallel to the identification of VOCs and VOSCs via GC analysis, the quantification of hydrogen sulphide, released in the stripped gas phase, was conducted by using an electrochemical sensor. The importance of monitoring this compound is correlated with its low odour threshold value (OTV), equal to 5.7 × 10^−4^ mg/m^3^ [36]. The H_2_S concentration of the first samples of the examined crude oils is shown in Table S9 of the Supplementary Materials. Crude G had the highest content, with 14 mg/Nm^3^, and crude C had the lowest, with 0.46 mg/Nm^3^.

### 2.3. Odour Characterisation: Correlation Between Chemical Composition and Odour Potential

Thanks to the identification and quantification of single compounds present in the gaseous samples, it was possible to investigate the mass contribution in terms of the chemical concentration (expressed in mg/m^3^) and odour contribution of the detected VOCs. Therefore, a comparison of the mass and the odour contributions of the hydrocarbons and sulphur compounds, respectively, was carried out. To evaluate the odour contribution of each single compound, the odour activity value (OAV_i_) of every single compound was calculated [37,38,39]. This parameter represents a first approximation of a compound’s contribution to the odour potential of a gaseous sample, and it can be calculated as follows:

(1) $${\mathrm{OAV}}_{i}=\frac{C_{i}}{{\mathrm{OTV}}_{i}}$$

where C_i_ is the concentration of a detected compound I, and OTV_i_ is its odour threshold value.

**Figure 5:**
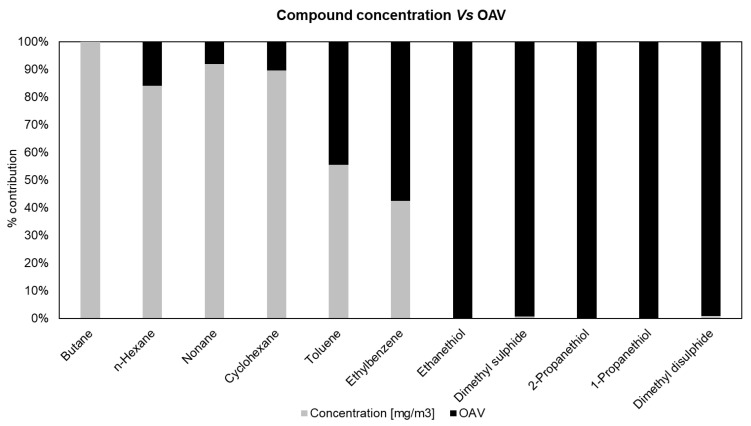
Percentage contribution of concentration (mg/m^3^) and OAV of main hydrocarbons and sulphur compounds for the first sample of crude oil A.

Therefore, for the first sample of every type of crude oil, the calculation of the OAVs for each detected compound was conducted. For the sake of brevity, the OAV results of crude oil A were taken as an illustrative case. In Figure 5, the OAV values, together with the mass concentration of some significant compounds detected in the first sample (after 30 min of bubbling), are reported as follows: six hydrocarbon compounds, both aliphatic and aromatic, amongst the most concentrated (i.e., butane, n-hexane, nonane, cyclohexane, toluene, ethylbenzene), and the five sulphur compounds (i.e., ethanethiol, dimethyl sulphide, 1-propanethiol, 2-propanethiol, dimethyl disulphide).

The most significant compounds in terms of odour (high OAV values, black columns in Figure 5) are mainly sulphur compounds, which, however, are in the minority in terms of concentration (low concentration values, grey columns in Figure 5). In contrast, hydrocarbons, the main constituents of the gaseous mixture (high concentration values, grey columns in Figure 5), exhibit a negligible odour contribution. From this evidence, it appears possible to state that the observed difference in odour concentration for the different crude samples (Figure 2) can therefore be correlated to the presence of these sulphur compounds. Indeed, crudes A, F, and G, which presented peaks in the PFPD chromatograms (Figure 4), were also the most odorous types, with a C_od_ always higher than 10^6^ ou_E_/m^3^ (Figure 2).

A further investigation, carried out to better investigate the odour potential of crude oils, was the search for a relationship among odour concentration, H_2_S concentration, and the percentage of sulphur content in the liquid matrix (%S). Sulphur content (%S) represents the quantity of sulphur present in the crude oil, and this parameter describes the weight percent of sulphur in the crude oil. The higher the %S value, the richer it is in sulphur, and therefore, the more sour-smelling the crude oil. It is the norm that the matrix emitting the most H_2_S or in general, containing the largest proportion of sulphur (expressed as % m/m), is likely the most odorous. 

**Figure 6:**
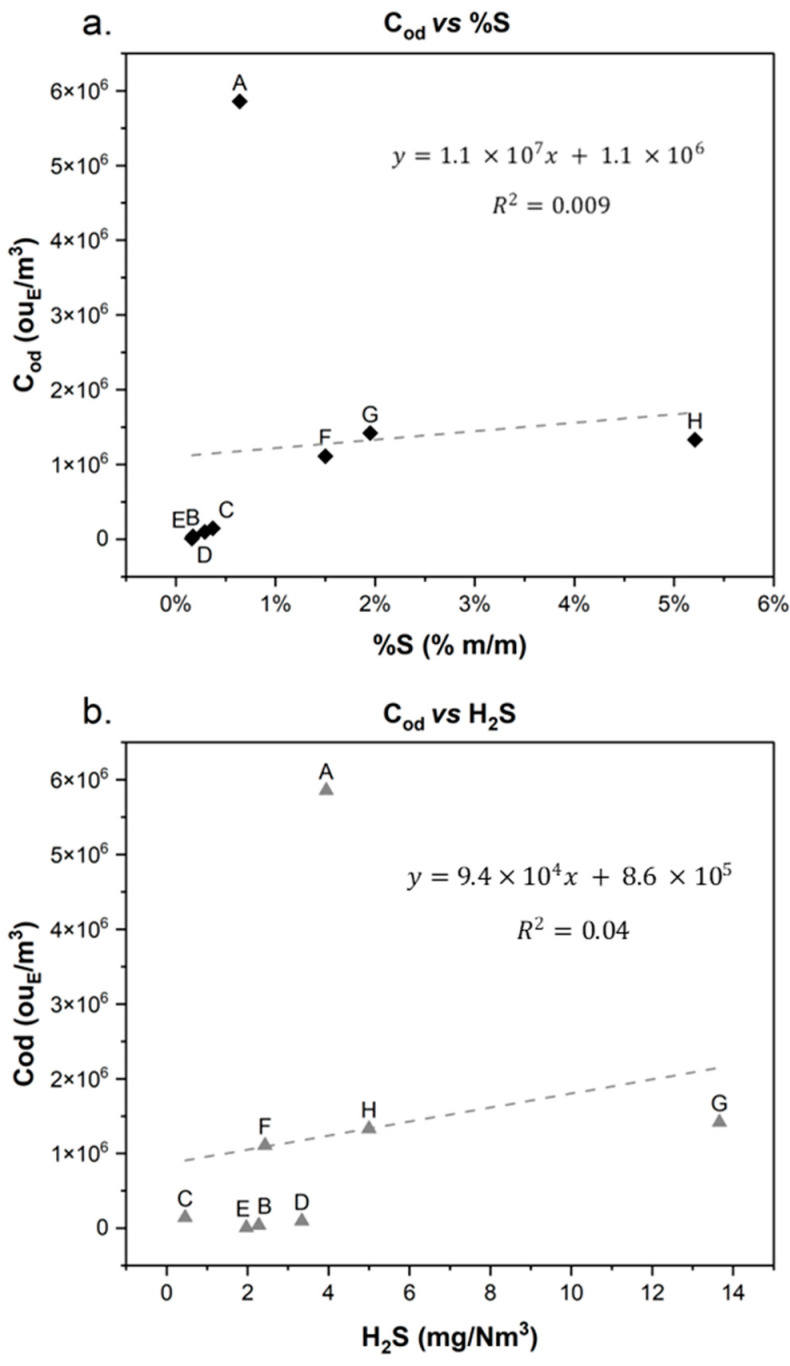
Trend of odour concentration as a function of %S (**a**) and as a function of H_2_S (**b**).

In the following diagrams, the odour concentration of each crude sample was reported as a function of the percentage of sulphur content (Figure 6a) and as a function of H_2_S measured in the first minutes (Figure 6b).

Figure 6 clearly shows that crude oil A significantly alters the linear correlations related to odorous potential. It was identified as the most odorous sample, with a C_od_ for the first sample (30 min) equal to 5.9 × 10^6^ ou_E_/m^3^ but corresponding to a relatively low sulphur content (%S = 0.64%) and H_2_S concentration (3.9 mg/Nm^3^).

**Figure 7:**
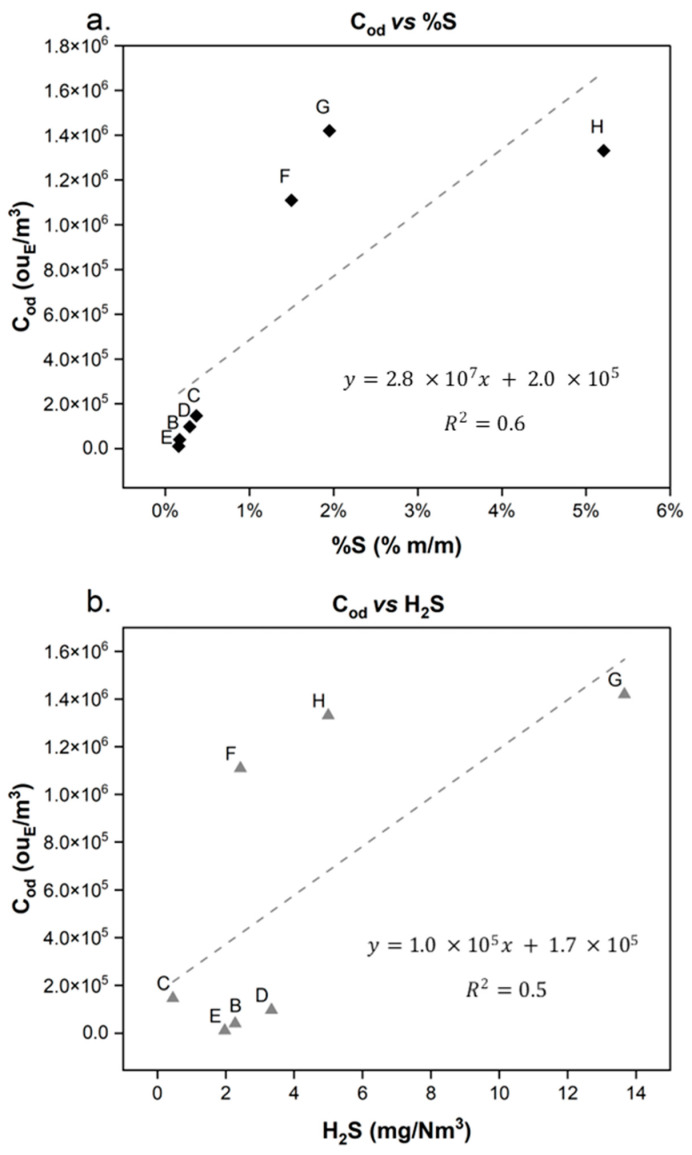
Trend of odour concentration as a function of %S (**a**) and as a function of H_2_S (**b**) (excluding crude oil A).

By removing crude oil A (Figure 7) from the graph, a linear correlation between odour concentration and the percentage of sulphur in the liquid could be observed. Also, the correlation between C_od_ and H_2_S appears to be slightly linear. Thus, as a first approximation, the sulphur content of crude oil can provide preliminary information about its odorous property, while the concentration of hydrogen sulphide seems to be a less effective indicator for predicting odour concentration.

To investigate the anomalous trend of the C crude, the VOSCs observed in the vapour of sample A was investigated in-depth. Indeed, as previously mentioned, crude C was one of the most odorous crude oils. Therefore, a detailed comparison, in terms of VOSCs, was conducted for the three most odorous crude oils (crude oils A, F, G, and H, with C_od_ values higher than 10^6^ ou_E_/m^3^, Figure 2). The VOSCs detected in the three crude oils, with their OTVs, are reported in Table 1. In Table S10, the molecule structure of the VOSCs detected in the samples A, F, G, and H are reported.

From the speciation of the samples, as previously highlighted in Figure 4, crude oil A presents different types of VOSCs compared to those in the other crude oils analysed. Compared with other crude oil emissions, crude oil A presents the most variable range of sulphur compounds (Figure 4 and Table 1). The additional sulphur compounds observed in crude oil A (ethanethiol, 1-propanethiol, dimethyl disulphide, methyl ethyl disulphide), characterised by a lower OTV (as reported in Table 1), could lead to a significant increase in odour potential, thus explaining the observed peak odour concentration value (5.9 × 10^6^ ou_E_/m^3^), justifying the observed outlier and highlighting the importance of VOSCs in crude oil odour potential. Therefore, to effectively assess the odour potential of crude oils, the presence of VOSCs within the vapour is predominant in terms of odour potential, compared to the effects of other known properties (H_2_S content or %S present in the liquid matrix).

## 3. Materials and Methods

### 3.1. Experimental Setup

In this study, eight liquid crude oil samples, with different worldwide origins, were investigated. Each was referenced with a different letter (A to H). To simulate gaseous emissions from crude oils, a laboratory method developed and described in a previous paper [7] was used for the characterisation of diffuse emissions from external floating roof tanks in which crude oils are generally stored. The experiment was based on the bubbling of the crude oil liquid sample with a controlled nitrogen flux, generating a gaseous outlet enriched with the stripped compounds. To evaluate the concentration trend during the time, for each crude oil, the gaseous emissions were sampled with Nalophan^TM^ bags (Tillmanns S.p.A., Milan, Italy) at different flushing times: 30 min, 8 h, 24 h, 192 h (8 days), and 576 h (24 days) from the beginning of the fluxing. 

Each obtained gaseous sample was analysed both in chemical and olfactometric terms, as described in the following paragraphs.

### 3.2. Determination of Odour Concentration (C_od_): Dynamic Olfactometry

Dynamic olfactometry is the standardised method for measuring the odour concentration (C_od_) of gaseous emissions using the human sense of smell. It involves the collection of gaseous samples, the dilution with odourless air, and the presentation of the sample to a standardised panel at increasing concentrations until the odour is detected. The outcome of the analysis is the odour concentration of the sample, expressed in European odour units per cubic meter (ou_E_/m^3^). According to the standard, it is defined as the number of times the gaseous sample is diluted with neutral air before its odour is statistically no longer perceivable [40]. The analyses were performed using an olfactometer (TO8, Ecoma GmbH, Kiel, Germany), based on the “Yes/No” method, involving four examiners during the olfactometric analysis. The panel involved is selected through tests of olfactory sensitivity concerning the standardised compound n-butanol (60 ppm in nitrogen SAPIO Group, Caponago, Italy), according to the criteria prescribed by the technical standard.

### 3.3. Determination of Chemical Composition

The gaseous samples were analysed using different analytical instrumentations, complementary with each other, to obtain the most complete quantitative and qualitative characterisation of the investigated gaseous samples.

#### 3.3.1. Determination of TVOC: Flame Ionization Detector (FID)

A flame ionization detector (FID) instrument was used to measure the total volatile organic carbon (TVOC) in the gaseous samples. The equipment used experimentally was the Total Hydrocarbon Analyzer RS 55-T (Ratfisch, Poing, Germany), in compliance with the standard [41]. The instrument was calibrated with specific gas bottles of propane (SAPIO Group, Caponago, Italy). The concentration of propane, expressed in ppm and measured during the FID analysis, can be easily converted to TVOC, as follows (2):

(2) $$C_{TVOC}=C_{C3H8}\times \frac{3\times M_{C}}{V_{M}}=C_{C3H8}\times \frac{3\times 12}{22.4}$$

where *C_TVOC_* is the TVOC concentration of the samples (expressed as mg_C_/Nm^3^), *C_C_*_3*H*8_ is the FID output (in ppm of propane, C_3_H_8_) to be converted, *M_C_* is the molecular weight of the carbon atom (12 g/mol), and *V_M_* is the normal volume of air (22.4 NL/mol) at 273.15 K and 101.3 kPa.

#### 3.3.2. Quantification and Identification of VOCs: GC-FID/MS/PFPD

To obtain the quantification and identification of single VOCs present in the gaseous samples, analyses using a gas chromatograph (GC, Agilent, model 8890, Folsom, CA, USA) coupled with three detectors (a single quadrupole mass spectrometer detector, MS Agilent 5977B MSD, Folsom, CA, USA; an FID; and a pulsed flame photometric detector, PFPD, OI 5883, OI Analytics, TX, USA) were conducted. The analytical setup was described in detail in a previous study [42,43,44] and is briefly described below. By using a calibrated pump (Markes, Air Server-xr, Bridgend, UK), air samples were collected directly from the bags, utilized for the olfactometric measurements, and submitted to thermal desorption (Unity-xrTM, Markes International Ltd., Bridgend, UK) to preconcentrate the analytes present in the gaseous samples. The gas was then sent directly to a cold trap (TO-15/TO-17 air toxics, compatible with the simultaneous analysis of analytes from C_2/3_ to C_30/32_, Markes International Ltd.), maintained at −27 °C. The thermal desorption was conducted by heating the trap from −27 °C to 300 °C, and the compounds were sent by hot transfer line into the capillary column (DB-Sulphur SCD, 60 m × 0.320 mm × 4.20 μm, Agilent J&W, Folsom, CA, USA).

The quantitative estimation of analytes was conducted by GC-FID signals, using an external standard calibration method. Standard cylinders (SIAD SpA, Bergamo, Italy), containing various odorous and/or volatile compounds at known concentrations, were used to obtain molecule-specific response factors. Thanks to the PFPD detector, it was possible to specifically detect and quantify volatile organic sulphur compounds (VOSCs), notoriously known for their strong odour contributions [45].

The VOCs were identified using an MS detector by comparing the spectra with the NIST20 database (NIST/EPA/NIH Mass Spectral Library, Version 2.4, 25 March 2020).

#### 3.3.3. Hydrogen Sulphide Quantification: Electrochemical Sensor

In addition to the GC analysis, a specific analysis was performed to determine hydrogen sulphide levels. To evaluate the H_2_S content of the various gaseous samples, a specific electrochemical sensor (Optima 7 biogas analyser, MRU GmbH, Neckarsulm, Germany) was used. This instrument’s measuring range is 0–2800 mg/m^3^ H_2_S.

## 4. Conclusions

The handling of crude oil is one of the main sources of VOCs and odour in the oil refining industry. Despite the importance of this topic, only few scientific papers are available regarding the detailed chemical characterisation of crude oil vapour emissions. In addition, no studies dealing with the odour potential of these emissions are available. Therefore, this paper aimed to analyse, both chemically and via dynamic olfactometry, the gaseous and vapour emission of eight different crude oils, investigating their chemical composition and assessing their odour potential. The gaseous samples were obtained by a controlled-bubbling of the different liquid samples.

It was found that odour and TVOC concentrations of gaseous emissions have a similar decreasing trend over the bubbling time, according to the volatility trend, i.e., very volatile VOCs are emitted in the early stages of bubbling and in higher concentrations that gradually decrease. Focusing the analysis on the first, and richest, sample (i.e., 30 min of bubbling), C_od_ was very variable amongst crudes compared to the results for TVOC. As observed from GC–MS/FID/PFPD analysis, the emission of VOCs does not drastically influence the odour concentration (with a similar emissive profile for all tested crude oils), but the presence of sulphur compounds (i.e., mercaptans and sulphides) in the gaseous emissions of specific crude oils explains the variability of the observed C_od_. VOSCs characterised by very low OTVs (i.e., higher OAVs) could be associated with high values of C_od_ measured for these crude oils. Indeed, the crudes with the highest C_od_ values (crudes A, F, G, and H, with a C_od_ with an order of magnitude of 10^6^ ou_E_/m^3^) are characterised by the widest presence of VOSCs. From the monitoring of H_2_S and considering the sulphur content (%S) in the crude oil, it emerged that the odour concentration of this kind of matrix is generally proportional to the sulphur content, and less significantly, to the H_2_S. This general trend is, however, heavily skewed considering the crude oil A. Indeed, this crude is the most odorous, even if it does not exhibit the highest content of sulphur (%S) and H_2_S. This variation can be linked to its release of VOSCs; these compounds are characterised by a very low OTV (on the order of µg/m^3^).

As a general outcome, it clearly appears that crude oils, even if considered similar from a physical and elemental point of view, are actually very different in terms of the chemical composition of vapours. The derived diffuse odorous emissions, and therefore, their potential impact, could be very different depending on the raw material, and the crude oils’ odorous potential is correlated, based on the results obtained, more with the presence of VOSC than with other physical properties (known a priori) of the crude. This result is extremely important for addressing, in greater detail, the issue of odour emissions from the storage/movement of crude oil. 

Different future developments of this work should be explored. Firstly, an improved chemical composition analysis of crude oil vapours could be conducted by integrating a volatile nitrogen compound detector. Moreover, this analysis could be expanded to a larger number of crude oil samples. Finally, possible strategies to mitigate the odour impact of crude oil emissions could be investigated, based on the results of this study. Potential approaches may include optimizing storage and handling conditions, implementing advanced vapour recovery systems, or developing tailored treatment methods to reduce the concentration of the most odour-relevant compounds.

## Figures and Tables

**Table 1:** Detected sulphur compounds (highlighted with ●) for crudes A, F, G, and H. The points indicate the presence of a compound.

Detected Sulphur Compound	OTV [mg/m^3^]	A	F	G	H
Methanethiol	1.4 × 10^−4^		●	●	●
Ethanethiol	2.2 × 10^−5^	●			●
Dimethyl sulphide	7.6 × 10^−3^	●	●	●	
2-Propanethiol	1.9 × 10^−5^	●	●	●	●
1-Propanethiol	4.0 × 10^−5^	●			
Dimethyl disulphide	8.5 × 10^−3^	●			
Methyl Ethyl Disulphide	-	●			

## Data Availability

The data used are confidential.

## Supplementary Materials

The following supporting information can be downloaded at: https://www.mdpi.com/article/10.3390/molecules30051136/s1, Table S1: Chemical species attribution for crude A—30 min. Table S2: Chemical species attribution for crude B—30 min; Table S3: Chemical species attribution for crude C—30 min; Table S4: Chemical species attribution for crude D—30 min; Table S5: Chemical species attribution for crude E—30 min; Table S6: Chemical species attribution for crude F—30 min; Table S7: Chemical species attribution for crude G—30 min; Table S8: Chemical species attribution for crude H—30 min; Table S9: H2S concentration of first 30 min, %S, and first sample C_od_ for each crude; Table S10. Molecule structures of detected VOSCs in the samples A, F, G, and H.

## Abbreviations

%S	mass sulphur content
BTEX	benzene, toluene, ethylbenzene, xilenes
C_od_	odour concentration
FID	flame ionization detector
GC	gas chromatography
GC-MS	gas chromatography–mass spectrometry
MS	mass spectrometry
OAV	odour activity value
OTV	odour threshold value
PFPD	pulsed flame photometric detector
TVOC	total volatile organic carbon
VOC	volatile organic compound
VOSC	volatile organic sulphur compound
