# Supplementary material for: Olfactometric and Chemical Characterisation of Gaseous Emission from Crude Oils
Source: Molecules. 2025 Mar 1;30(5):1136. doi: 10.3390/molecules30051136 (PMC11901996; doi:10.3390/molecules30051136)
Supplement: Supplementary file 1 [file molecules-30-01136-s001.zip › molecules-3458065-supplementary.pdf]

Supplementary Material

Olfactometric and Chemical Characterization of Gaseous Emission from Crude Oils

E. Polvara[1], *V. Legnani*[1], *M. Invernizzi*[1,*], *S. Sironi*[1]

[1] Poiltecnico di Milano, Department of Chemistry, Materials and Chemical Engineering "Giulio Natta", Piazza Leonardo da Vinci 32, 20133, Milano, Italy

*Corresponding author; email: marzio.invernizzi@poilmi.it

Crude oil A (30 min)					
RT [min]	Compound	CAS	Detector	Concentration [mg/m³]	OAV
3.323	Propane	74-98-6	FID	619.18	2.29E-01
3.995	Isobutane	75-28-5	FID	9981.61	
4.483	Butane	106-97-8	FID	52118.22	1.83E+01
4.722	Neopentane	463-82-1	FID	436.47	
5.959	Butane, 2-methyl-	78-78-4	FID	45528.96	1.19E+04
6.489	Ethanethiol	75-08-1	PFPD	19.84	8.97E+05
6.574	Pentane	109-66-0	FID	38327.69	9.28E+03
6.931	Dimethyl sulphide	75-18-3	PFPD	37.42	4.91E+03
7.608	Butane, 2,2-dimethyl-	75-83-2	FID	654.71	9.29E+00
7.988	2-Propanethiol	75-33-2	PFPD	68.50	3.66E+06
8.562	Pentane, 2-methyl-	107-83-5	FID	17252.15	6.99E+02
9.044	Pentane, 3-methyl-	96-14-0	FID	4524.45	1.44E+02
9.173	Unknown sulphur compound		PFPD	5.43	
9.544	n-Hexane	110-54-3	FID	14908.10	2.82E+03
9.577	1-Propanethiol	107-03-9	PFPD	11.78	2.91E+05
9.755	Unknown sulphur compound		PFPD	9.45	
10.41	Butane, 2,2,3-trimethyl-	464-06-2	FID	595.47	
10.622	Cyclopentane, methyl-	96-37-7	FID	5333.53	9.11E+02
10.901	Pentane, 2,2-dimethyl-	590-35-2	FID	100.93	6.48E-01
11.456	Benzene	71-43-2	FID	759.30	8.80E+01
11.557	Sulfurous acid, hexyl pentyl ester	959059-25-7	PFPD	180.08	
11.811	Cyclohexane	110-82-7	FID	5236.34	6.09E+02
11.965	Pentane, 2,3-dimethyl-	565-59-3	FID	1607.85	8.72E+01
12.127	Hexane, 3-methyl-	589-34-4	FID	2437.39	7.08E+02
12.523	Cyclopentane, 1,3-dimethyl-	2453-00-1	FID	1082.65	
12.715	Cyclopentane, 1,2-dimethyl-	2452-99-5	FID	1764.45	
12.898	Heptane	142-82-5	FID	4351.27	1.58E+03

Crude oil A (30 min)					
RT [min]	Compound	CAS	Detector	Concentration [mg/m³]	OAV
13.979	Cyclohexane, methyl-	108-87-2	FID	2968.11	4.93E+03
14.135	Hexane, 2,4-dimethyl-	589-43-5	FID	526.58	
14.188	Dimethyl disulphide	624-92-0	PFPD	28.11	3.32E+03
14.288	Cyclopentane, ethyl-	1640-89-7	FID	371.81	
14.525	Cyclopentane, 1,2,4-trimethyl-	2815-58-9	FID	353.05	
14.785	Cyclopentane, 1,2,3-trimethyl-	2815-57-8	FID	497.53	
15.103	Toluene	108-88-3	FID	1246.16	1.00E+03
15.185	Heptane, 2-methyl-	592-27-8	FID	4729.19	9.20E+03
15.476	Heptane, 3-methyl-	589-81-1	FID	555.39	7.93E+01
15.912	Cyclohexane, 1,4-dimethyl-	589-90-2	FID	839.45	
16.316	Octane	111-65-9	FID	892.30	1.12E+02
16.647	Cyclohexane, 1,2-dimethyl-	583-57-3	FID	269.57	
16.841	Cyclohexane, 1,3-dimethyl-	591-21-9	FID	86.92	
17.17	Heptane, 2,4-dimethyl-	2213-23-2	FID	125.71	
17.348	Methyl ethyl disulphide	20333-39-5	PFPD	16.37	
17.35	Octane, 2-methyl-	3221-61-2	FID	1044.09	
17.628	Unknown		FID	100.81	
17.809	Cyclohexane, ethyl-	1678-91-7	FID	220.10	
17.992	Cyclohexane, 1,1,3-trimethyl-	3073-66-3	FID	291.89	
18.319	Ethylbenzene	100-41-4	FID	133.69	1.81E+02
18.491	Octane, 4-methyl-	2216-34-4	FID	518.93	
18.581	p+m-Xylene	106-42-3	FID	323.36	1.28E+03
18.74	Octane, 3-methyl-	2216-33-3	FID	457.82	
19.404	o-Xylene	95-47-6	FID	212.15	1.29E+02
19.577	Nonane	111-84-2	FID	413.69	3.58E+01
20.273	Unknown		FID	40.72	
20.443	Unknown		FID	21.09	

Crude oil A (30 min)					
**RT [min]**	**Compound**	**CAS**	**Detector**	**Concentration [mg/m^3]**	**OAV**
20.739	Octane, 2,6-dimethyl-	2051-30-1	FID	421.23	
20.943	Unknown		FID	47.03	
21.517	Benzene, 1-ethyl-3-methyl-	620-14-4	FID	127.09	1.44E+03
22.624	Decane	124-18-5	FID	88.75	2.46E+01

Table S1. Chemical species attribution Crude A – 30 minutes.

Crude oil B (30 min)					
RT [min]	Compound	CAS	Detector	Concentration [mg/m^3]	OAV
3.286	Propane	74-98-6	FID	2506.86	9.27E-01
3.967	Isobutane	75-28-5	FID	5961.32	
4.465	Butane	106-97-8	FID	20441.60	7.17E+00
4.703	Neopentane	463-82-1	FID	305.26	
5.944	Butane, 2-methyl-	78-78-4	FID	19105.13	4.98E+03
6.561	Pentane	109-66-0	FID	17234.01	4.17E+03
7.596	Butane, 2,2-dimethyl-	75-83-2	FID	999.21	1.42E+01
8.549	Pentane, 2-methyl-	107-83-5	FID	9044.17	3.67E+02
9.035	Pentane, 3-methyl-	96-14-0	FID	1977.55	6.30E+01
9.536	n-Hexane	110-54-3	FID	8208.89	1.55E+03
10.404	Butane, 2,2,3-trimethyl-	464-06-2	FID	916.77	
10.616	Cyclopentane, methyl-	96-37-7	FID	5012.26	8.57E+02
10.892	Pentane, 2,2-dimethyl-	590-35-2	FID	212.51	1.36E+00
11.451	Benzene	71-43-2	FID	924.90	1.07E+02
11.806	Cyclohexane	110-82-7	FID	5242.23	6.09E+02
11.957	Pentane, 2,3-dimethyl-	565-59-3	FID	766.60	4.16E+01
12.122	Hexane, 3-methyl-	589-34-4	FID	1364.39	3.96E+02
12.516	Cyclopentane, 1,3-dimethyl-	2453-00-1	FID	659.63	
12.704	Cyclopentane, 1,2-dimethyl-	2452-99-5	FID	1098.36	
12.887	Heptane	142-82-5	FID	2843.32	1.04E+03
13.699	Pentane, 2,2,4-trimethyl-	540-84-1	FID	121.29	3.87E+01
13.954	Cyclohexane, methyl- + Hexane, 2,4-dimethyl-		FID	2900.10	
14.262	Cyclopentane, ethyl-	1640-89-7	FID	238.71	
14.495	Cyclopentane, 1,2,4-trimethyl-	2815-58-9	FID	228.98	
14.758	Cyclopentane, 1,2,3-trimethyl-	2815-57-8	FID	252.86	
15.068	Toluene + Heptane, 2-methyl-		FID	1624.17	

Crude oil B (30 min)					
RT [min]	Compound	CAS	Detector	Concentration [mg/m³]	OAV
15.439	Heptane, 3-methyl-	589-81-1	FID	379.33	5.41E+01
15.873	Cyclohexane, 1,4-dimethyl-	589-90-2	FID	685.95	
16.276	Octane	111-65-9	FID	613.11	7.72E+01
16.607	Cyclohexane, 1,2-dimethyl-	583-57-3	FID	286.05	
16.801	Cyclohexane, 1,3-dimethyl-	591-21-9	FID	105.92	
17.13	Heptane, 2,4-dimethyl-	2213-23-2	FID	94.32	
17.313	Octane, 2-methyl-	3221-61-2	FID	430.10	
17.592	Heptane, 2,5-dimethyl-	2216-30-0	FID	427.30	
17.771	Cyclohexane, ethyl-	1678-91-7	FID	260.53	
17.957	Cyclohexane, 1,1,3-trimethyl-	3073-66-3	FID	142.68	
18.285	Ethylbenzene	100-41-4	FID	98.91	1.34E+02
18.456	Octane, 4-methyl-	2216-34-4	FID	462.77	
18.545	p+m-Xylene	106-42-3	FID	241.81	9.60E+02
18.709	Octane, 3-methyl-	2216-33-3	FID	244.32	
19.047	Pentalene, octahydro-	694-72-4	FID	41.00	
19.374	o-Xylene	95-47-6	FID	164.90	9.99E+01
19.551	Nonane	111-84-2	FID	311.78	2.70E+01
20.25	Cyclohexane, 1-ethyl-2-methyl-	3728-54-9	FID	82.67	
20.93	Cyclohexane, propyl- + Heptane, 3-ethyl-2-methyl-		FID	81.72	
21.5	Benzene, 1-ethyl-3-methyl-	620-14-4	FID	97.90	1.11E+03
22.623	Decane	124-18-5	FID	62.41	1.73E+01

Table S2. Chemical species attribution Crude B – 30 minutes.

Crude oil C (30 min)					
RT [min]	Compound	CAS	Detector	Concentration [mg/m³]	OAV
3.249	Propane	74-98-6	FID	2207.14	8.16E-01
3.928	Isobutane	75-28-5	FID	6409.78	
4.428	Butane	106-97-8	FID	36553.07	1.28E+01
4.668	Neopentane	463-82-1	FID	236.43	
5.919	Butane, 2-methyl-	78-78-4	FID	31795.77	8.29E+03
6.54	Pentane	109-66-0	FID	34781.06	8.42E+03
7.581	Butane, 2,2-dimethyl-	75-83-2	FID	819.39	1.16E+01
8.544	Pentane, 2-methyl-	107-83-5	FID	15993.37	6.48E+02
9.027	Pentane, 3-methyl-	96-14-0	FID	3699.06	1.18E+02
9.531	n-Hexane	110-54-3	FID	16107.16	3.05E+03
10.401	Butane, 2,2,3-trimethyl-	464-06-2	FID	1272.63	
10.614	Cyclopentane, methyl-	96-37-7	FID	7589.81	1.30E+03
10.892	Pentane, 2,2-dimethyl-	590-35-2	FID	173.42	1.11E+00
11.451	Benzene	71-43-2	FID	1208.34	1.40E+02
11.809	Cyclohexane	110-82-7	FID	11155.83	1.30E+03
11.96	Pentane, 2,3-dimethyl-	565-59-3	FID	1003.12	5.44E+01
12.128	Hexane, 3-methyl-	589-34-4	FID	3314.25	9.63E+02
12.524	Cyclopentane, 1,3-dimethyl-	2453-00-1	FID	1738.61	
12.714	Cyclopentane, 1,2-dimethyl-	2452-99-5	FID	3321.95	
12.899	Heptane	142-82-5	FID	7032.79	2.56E+03
13.725	Pentane, 2,2,4-trimethyl-	540-84-1	FID	276.88	8.85E+01
13.981	Cyclohexane, methyl- + Hexane, 2,4-dimethyl-		FID	6969.76	
14.29	Cyclopentane, ethyl-	1640-89-7	FID	331.33	
14.528	Cyclopentane, 1,2,4-trimethyl-	2815-58-9	FID	666.60	
14.79	Cyclopentane, 1,2,3-trimethyl-	2815-57-8	FID	526.71	
15.106	Toluene	108-88-3	FID	2747.69	2.21E+03

Crude oil C (30 min)					
RT [min]	Compound	CAS	Detector	Concentration [mg/m³]	OAV
15.187	Heptane, 2-methyl-	592-27-8	FID	7879.83	1.53E+04
15.481	Heptane, 3-methyl-	589-81-1	FID	1160.46	1.66E+02
15.917	Cyclohexane, 1,4-dimethyl-	589-90-2	FID	2096.30	
16.323	Octane	111-65-9	FID	1445.52	1.82E+02
16.652	Cyclohexane, 1,2-dimethyl-	583-57-3	FID	572.90	
16.846	Cyclohexane, 1,3-dimethyl-	591-21-9	FID	267.34	
17.172	Heptane, 2,4-dimethyl-	2213-23-2	FID	233.44	
17.356	Octane, 2-methyl-	3221-61-2	FID	1201.76	
17.619	Heptane, 2,5-dimethyl-	2216-30-0	FID	1068.48	
17.814	Cyclohexane, ethyl-	1678-91-7	FID	596.88	
17.998	Cyclohexane, 1,1,3-trimethyl-	3073-66-3	FID	293.22	
18.33	Ethylbenzene	100-41-4	FID	132.74	1.80E+02
18.501	Octane, 4-methyl-	2216-34-4	FID	1257.33	
18.59	p+m-Xylene	106-42-3	FID	763.24	3.03E+03
18.753	Octane, 3-methyl-	2216-33-3	FID	670.69	
19.424	o-Xylene	95-47-6	FID	251.76	1.53E+02
19.599	Nonane	111-84-2	FID	630.48	5.46E+01
20.297	Cyclohexane, 1-ethyl-2-methyl-	3728-54-9	FID	124.70	
20.982	Cyclohexane, propyl-	1678-92-8	FID	138.93	
21.559	Benzene, 1-ethyl-3-methyl-	620-14-4	FID	149.43	1.69E+03
22.672	Decane	124-18-5	FID	83.43	2.31E+01

Table S3. Chemical species attribution Crude C – 30 minutes.

Crude oil D (30 min)					
RT [min]	Compound	CAS	Detector	Concentration [mg/m³]	OAV
3.291	Propane	74-98-6	FID	4942.83	1.83E+00
3.969	Isobutane	75-28-5	FID	5909.81	
4.466	Butane	106-97-8	FID	27003.22	9.47E+00
4.703	Neopentane	463-82-1	FID	124.92	
5.946	Butane, 2-methyl-	78-78-4	FID	23690.72	6.18E+03
6.564	Pentane	109-66-0	FID	26302.97	6.37E+03
7.599	Butane, 2,2-dimethyl-	75-83-2	FID	440.60	6.25E+00
8.555	Pentane, 2-methyl-	107-83-5	FID	11421.31	4.63E+02
9.038	Pentane, 3-methyl-	96-14-0	FID	2638.36	8.41E+01
9.54	n-Hexane	110-54-3	FID	12333.84	2.33E+03
10.407	Butane, 2,2,3-trimethyl-	464-06-2	FID	702.16	
10.619	Cyclopentane, methyl-	96-37-7	FID	5376.11	9.19E+02
10.902	Pentane, 2,2-dimethyl-	590-35-2	FID	150.98	9.70E-01
11.454	Benzene	71-43-2	FID	619.49	7.18E+01
11.81	Cyclohexane	110-82-7	FID	4924.08	5.72E+02
11.964	Pentane, 2,3-dimethyl-	565-59-3	FID	1247.64	6.77E+01
12.129	Hexane, 3-methyl-	589-34-4	FID	1801.83	5.23E+02
12.525	Cyclopentane, 1,3-dimethyl-	2453-00-1	FID	978.48	
12.715	Cyclopentane, 1,2-dimethyl-	2452-99-5	FID	1890.12	
12.898	Heptane	142-82-5	FID	4284.64	1.56E+03
13.979	Cyclohexane, methyl-	108-87-2	FID	3544.35	5.88E+03
14.288	Cyclopentane, ethyl-	1640-89-7	FID	238.57	
14.527	Cyclopentane, 1,2,4-trimethyl-	2815-58-9	FID	356.81	
14.791	Cyclopentane, 1,2,3-trimethyl-	2815-57-8	FID	465.52	
15.11	Toluene	108-88-3	FID	499.51	4.02E+02
15.189	Heptane, 2-methyl-	592-27-8	FID	4542.26	8.84E+03

Crude oil D (30 min)					
**RT [min]**	**Compound**	**CAS**	**Detector**	**Concentration [mg/m³]**	**OAV**
15.475	Heptane, 3-methyl-	589-81-1	FID	378.36	5.40E+01
15.912	Cyclohexane, 1,4-dimethyl-	589-90-2	FID	1135.05	
16.318	Octane	111-65-9	FID	893.07	1.12E+02
16.648	Cyclohexane, 1,2-dimethyl-	583-57-3	FID	435.03	
16.841	Cyclohexane, 1,3-dimethyl-	591-21-9	FID	131.34	
17.168	Heptane, 2,4-dimethyl-	2213-23-2	FID	183.12	
17.351	Octane, 2-methyl-	3221-61-2	FID	666.17	
17.633	Heptane, 2,5-dimethyl-	2216-30-0	FID	408.66	
17.807	Cyclohexane, ethyl-	1678-91-7	FID	306.50	
17.989	Cyclohexane, 1,1,3-trimethyl-	3073-66-3	FID	314.35	
18.315	Ethylbenzene	100-41-4	FID	131.10	1.78E+02
18.482	Octane, 4-methyl-	2216-34-4	FID	818.63	
18.569	p+m-Xylene	106-42-3	FID	108.23	4.30E+02
18.739	Octane, 3-methyl-	2216-33-3	FID	346.70	
19.079	3-Cyclopentyl-1-propanol	767-05-5	FID	44.41	
19.406	o-Xylene	95-47-6	FID	148.04	8.97E+01
19.577	Nonane	111-84-2	FID	476.65	4.13E+01
20.272	Cyclohexane, 1-ethyl-2-methyl-	3728-54-9	FID	99.36	
20.954	Cyclohexane, propyl-	1678-92-8	FID	158.09	
22.643	Decane	124-18-5	FID	82.84	2.30E+01

Table S4. Chemical species attribution Crude D – 30 minutes.

Crude oil E (30 min)					
RT [min]	Compound	CAS	Detector	Concentration [mg/m³]	OAV
3.31	Propane	74-98-6	FID	4148.17	1.53E+00
3.987	Isobutane	75-28-5	FID	6430.74	
4.483	Butane	106-97-8	FID	22199.92	7.78E+00
4.72	Neopentane	463-82-1	FID	232.18	
5.957	Butane, 2-methyl-	78-78-4	FID	23577.43	6.15E+03
6.571	Pentane	109-66-0	FID	23616.25	5.72E+03
7.604	Butane, 2,2-dimethyl-	75-83-2	FID	1189.65	1.69E+01
8.557	Pentane, 2-methyl-	107-83-5	FID	13772.21	5.58E+02
9.039	Pentane, 3-methyl-	96-14-0	FID	3196.52	1.02E+02
9.539	n-Hexane	110-54-3	FID	14995.69	2.84E+03
10.405	Butane, 2,2,3-trimethyl-	464-06-2	FID	1657.05	
10.616	Cyclopentane, methyl-	96-37-7	FID	6406.09	1.09E+03
10.896	Pentane, 2,2-dimethyl-	590-35-2	FID	416.26	2.67E+00
11.451	Benzene	71-43-2	FID	1190.98	1.38E+02
11.804	Cyclohexane	110-82-7	FID	6991.15	8.12E+02
11.956	Pentane, 2,3-dimethyl-	565-59-3	FID	1646.66	8.93E+01
12.12	Hexane, 3-methyl-	589-34-4	FID	2716.11	7.89E+02
12.515	Cyclopentane, 1,3-dimethyl-	2453-00-1	FID	1216.11	
12.703	Cyclopentane, 1,2-dimethyl-	2452-99-5	FID	1975.28	
12.885	Heptane	142-82-5	FID	6653.32	2.42E+03
13.699	Pentane, 2,2,4-trimethyl-	540-84-1	FID	286.28	9.15E+01
13.953	Cyclohexane, methyl- + Hexane, 2,4-dimethyl-		FID	4732.11	
14.259	Cyclopentane, ethyl-	1640-89-7	FID	346.21	
14.494	Cyclopentane, 1,2,4-trimethyl-	2815-58-9	FID	493.29	
14.756	Cyclopentane, 1,2,3-trimethyl-	2815-57-8	FID	501.66	
15.068	Toluene	108-88-3	FID	1229.94	9.89E+02

15.151	Heptane, 2-methyl-	592-27-8	FID	7070.15	1.38E+04
15.437	Heptane, 3-methyl-	589-81-1	FID	776.69	1.11E+02
15.869	Cyclohexane, 1,4-dimethyl-	589-90-2	FID	1644.48	
16.273	Octane	111-65-9	FID	1514.90	1.91E+02
16.602	Cyclohexane, 1,2-dimethyl-	583-57-3	FID	576.87	
16.798	Cyclohexane, 1,3-dimethyl-	591-21-9	FID	183.08	
17.001	Unknown		FID	55.72	
17.125	Heptane, 2,4-dimethyl-	2213-23-2	FID	231.74	
17.309	Octane, 2-methyl-	3221-61-2	FID	904.74	
17.58	Heptane, 2,5-dimethyl-	2216-30-0	FID	887.59	
17.768	Cyclohexane, ethyl-	1678-91-7	FID	495.27	
17.951	Cyclohexane, 1,1,3-trimethyl-	3073-66-3	FID	393.36	
18.282	Ethylbenzene	100-41-4	FID	196.46	2.66E+02
18.449	Octane, 4-methyl-	2216-34-4	FID	1571.64	
18.539	p+m-Xylene	106-42-3	FID	329.80	1.31E+03
18.705	Octane, 3-methyl-	2216-33-3	FID	673.14	
19.373	o-Xylene	95-47-6	FID	236.36	1.43E+02
19.547	Nonane	111-84-2	FID	908.35	7.87E+01
20.243	Cyclohexane, 1-ethyl-2-methyl-	3728-54-9	FID	174.57	
20.717	Unknown		FID	109.31	
20.924	Cyclohexane, propyl-	1678-92-8	FID	243.81	
21.5	Unknown		FID	104.95	
21.566	Benzene, 1-ethyl-3-methyl-	620-14-4	FID	67.00	7.57E+02
22.622	Decane	124-18-5	FID	174.35	4.83E+01

Table S5. Chemical species attribution Crude E – 30 minutes.

Crude oil F (30 min)					
RT [min]	Compound	CAS	Detector	Concentration [mg/m³]	OAV
3.17	Propane	74-98-6	FID	8250.41	3.05E+00
3.818	Isobutane	75-28-5	FID	10080.38	
4.3	Butane	106-97-8	FID	61247.18	2.15E+01
4.521	Methanethiol	74-93-1	PFPD	36.09	2.62E+05
5.773	Butane, 2-methyl-	78-78-4	FID	46753.49	1.22E+04
6.294	Dimethyl sulfide	75-18-3	PFPD	72.49	9.51E+03
6.38	Pentane	109-66-0	FID	55018.87	1.33E+04
7.417	Butane, 2,2-dimethyl-	75-83-2	FID	312.16	4.43E+00
7.8	2-Propanethiol	75-33-2	PFPD	63.46	3.40E+06
8.37	Pentane, 2-methyl-	107-83-5	FID	14245.54	5.77E+02
8.851	Pentane, 3-methyl-	96-14-0	FID	5164.19	1.65E+02
9.349	n-Hexane	110-54-3	FID	15317.22	2.90E+03
10.428	Cyclopentane, methyl-	96-37-7	FID	4097.11	7.00E+02
11.279	Benzene	71-43-2	FID	878.22	1.02E+02
11.643	Cyclohexane	110-82-7	FID	3739.65	4.35E+02
11.803	Pentane, 2,3-dimethyl-	565-59-3	FID	2621.84	1.42E+02
11.97	Hexane, 3-methyl-	589-34-4	FID	3299.34	9.58E+02
12.372	Cyclopentane, 1,3-dimethyl-	2453-00-1	FID	753.87	
12.568	Cyclopentane, 1,2-dimethyl-	2452-99-5	FID	1351.18	
12.757	Heptane	142-82-5	FID	4707.11	1.71E+03
13.842	Cyclohexane, methyl-	108-87-2	FID	2547.28	4.23E+03
14.005	Hexane, 2,4-dimethyl-	589-43-5	FID	546.31	
14.154	Cyclopentane, ethyl-	1640-89-7	FID	376.29	
14.395	Cyclopentane, 1,2,4-trimethyl-	2815-58-9	FID	217.85	
14.659	Cyclopentane, 1,2,3-trimethyl-	2815-57-8	FID	274.94	
14.975	Toluene	108-88-3	FID	890.44	7.16E+02
15.07	Heptane, 2-methyl-	592-27-8	FID	4400.21	8.56E+03
15.357	Heptane, 3-methyl-	589-81-1	FID	634.77	9.06E+01
15.787	Cyclohexane, 1,4-dimethyl-	589-90-2	FID	373.34	
16.204	Octane	111-65-9	FID	1110.28	1.40E+02
16.532	Cyclohexane, 1,2-dimethyl-	583-57-3	FID	158.47	
17.248	Octane, 2-methyl-	3221-61-2	FID	636.36	
17.531	2-Propyl-1-pentanol	58175-57-8	FID	294.20	
17.71	Cyclohexane, ethyl-	1678-91-7	FID	166.50	
17.896	Cyclohexane, 1,1,3-trimethyl-	3073-66-3	FID	202.71	
18.233	Ethylbenzene	100-41-4	FID	245.81	3.33E+02
18.401	Octane, 4-methyl-	2216-34-4	FID	967.81	
18.497	p+m-Xylene	106-42-3	FID	209.72	8.33E+02
18.658	Octane, 3-methyl-	2216-33-3	FID	651.10	
19.331	o-Xylene	95-47-6	FID	112.11	6.79E+01

Crude oil F (30 min)					
RT [min]	Compound	CAS	Detector	Concentration [mg/m³]	OAV
19.507	Nonane	111-84-2	FID	492.50	4.27E+01
20.196	2-Hexene, 3,4,4-trimethyl-	53941-19-8	FID	66.40	
20.679	Benzene, (1-methylethyl)-	98-82-8	FID	80.30	1.94E+03
20.885	Nonane, 3-methyl-	5911-04-6	FID	269.91	
21.475	Cyclopentane, 1-methyl-2-propyl-	3728-57-2	FID	115.53	
22.598	Decane	124-18-5	FID	987.47	2.74E+02
25.486	Undecane	1120-21-4	FID	184.47	3.32E+01
28.19	Dodecane	112-40-3	FID	252.39	3.29E+02

Table S6. Chemical species attribution Crude F – 30 minutes.

Crude oil G (30 min)					
RT [min]	Compound	CAS	Detector	mg/m³	OAV
3.284	Propane	74-98-6	FID	4398.96	1.63E+00
3.956	Isobutane	75-28-5	FID	14599.91	
4.452	Butane	106-97-8	FID	99987.62	3.51E+01
4.666	Methanethiol	74-93-1	PFPD	237.84	1.73E+06
5.94	Butane, 2-methyl-	78-78-4	FID	69026.58	1.80E+04
6.559	Pentane	109-66-0	FID	84899.21	2.06E+04
6.9	Dimethyl sulfide	75-18-3	PFPD	236.37	3.10E+04
7.9	2-Propanethiol	75-33-2	PFPD	99.41	5.32E+06
8.56	Pentane, 2-methyl-	107-83-5	FID	18291.96	7.41E+02
9.044	Pentane, 3-methyl-	96-14-0	FID	6773.96	2.16E+02
9.546	n-Hexane	110-54-3	FID	24967.39	4.72E+03
10.629	Cyclopentane, methyl-	96-37-7	FID	3109.05	5.31E+02
11.464	Benzene	71-43-2	FID	1578.95	1.83E+02
11.81	Hexane, 2-methyl-	591-76-4	FID	16439.84	9.55E+03
11.966	Pentane, 2,3-dimethyl-	565-59-3	FID	3643.33	1.98E+02
12.126	Hexane, 3-methyl-	589-34-4	FID	3945.49	1.15E+03
12.516	Cyclopentane, 1,3-dimethyl-	2453-00-1	FID	692.89	
12.711	Cyclopentane, 1,2-dimethyl-	2452-99-5	FID	989.03	
12.891	Heptane	142-82-5	FID	7733.68	2.82E+03
13.957	Cyclohexane, methyl-	108-87-2	FID	2086.32	3.46E+03
15.077	Toluene + Heptane, 2-methyl-		FID	2845.97	
15.436	Heptane, 3-methyl-	589-81-1	FID	803.42	1.15E+02
15.868	Cyclohexane, 1,4-dimethyl-	589-90-2	FID	277.18	
16.272	Octane	111-65-9	FID	1601.01	2.02E+02
18.702	Octane, 3-methyl-	2216-33-3	FID	1418.47	
19.553	Nonane	111-84-2	FID	709.72	6.15E+01

Table S7. Chemical species attribution Crude G – 30 minutes.

Crude oil H (30 min)					
**RT [min]**	**Compound**	**CAS**	**Detector**	**mg/m³**	**OAV**
3.304	Propane	74-98-6	FID	5176.09	1.91E+00
3.960	Isobutane	75-28-5	FID	8577.90	
4.460	Butane	106-97-8	FID	62361.75	2.19E+01
4.676	Methanethiol	74-93-1	PFPD	49.66	3.61E+05
5.923	Butane, 2-methyl-	78-78-4	FID	49153.58	1.28E+04
6.4	Ethanethiol	75-08-1	PFPD	261.43	1.18E+07
6.599	Pentane	109-66-0	FID	25097.46	6.07E+03
6.98	2-Propanethiol	75-33-2	PFPD	169.51	9.07E+06
8.6	Pentane, 2-methyl-	107-83-5	FID	11949.27	4.84E+02
9.054	Pentane, 3-methyl-	96-14-0	FID	5434.55	1.73E+02
9.55	n-Hexane	110-54-3	FID	14035.35	2.65E+03
10.6	Cyclopentane, methyl-	96-37-7	FID	3246.85	5.55E+02
11.465	Benzene	71-43-2	FID	806.57	9.35E+01
11.7	Pentane, 3-ethyl-2,4-dimethyl-	1068-87-7	FID	2858.12	
12	Pentane, 2,3-dimethyl-	565-59-3	FID	830.87	4.51E+01
12.13	Hexane, 3-methyl-	589-34-4	FID	2746.44	7.98E+02
12.523	Cyclopentane, 1,3-dimethyl-	2453-00-1	FID	769.87	
12.722	Cyclopentane, 1,2-dimethyl-	2452-99-5	FID	1160.97	
12.8	Heptane	142-82-5	FID	4812.42	1.75E+03
13.844	Cyclohexane, methyl-	108-87-2	FID	1519.64	2.52E+03
14.155	Cyclopentane, ethyl-	1640-89-7	FID	308.19	
14.402	Cyclopentane, 1,2,4-trimethyl-	2815-58-9	FID	178.14	
14.667	Cyclopentane, 1,2,3-trimethyl-	2815-57-8	FID	258.34	
15.0	Toluene	108-88-3	FID	1938.41	1.56E+03

Table S8. Chemical species attribution Crude H – 30 minutes.

Crude oil	H_2S [mg/Nm^3]	S% [m/m]	C_{od} [ou$_E$/m^3]
A	3.95	0.64%	5.9E+06
B	2.28	0.17%	4.1E+04
C	0.46	0.37%	1.46E+05
D	1.97	0.16%	7.68E+03
E	3.34	0.29%	9.74E+04
F	2.43	1.5%	1.11E+06
G	13.66	1.95%	1.42E+06
H	7.01		

Table S9. H_2S concentration of first 30 minutes, %S and first sample C_{od} for each crude.

Detected sulphur compound	Molecule structures
Methanethiol	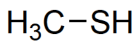
Ethanethiol	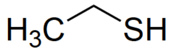
Dimethyl sulphide	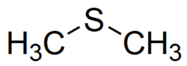
2-Propanethiol	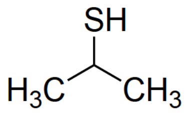
1-Propanethiol	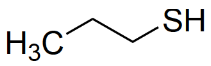
Dimethyl disulphide	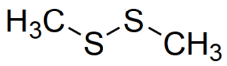
Methyl Ethyl Disulphide	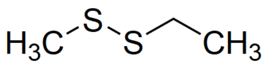

Table S10. Molecule structures of detected VOSCs in the samples A, F, G and H.
